# Terminal Phase Components of the Clotting Cascade in Patients with End-Stage Renal Disease Undergoing Hemodiafiltration or Hemodialysis Treatment

**DOI:** 10.3390/ijms21228426

**Published:** 2020-11-10

**Authors:** Krisztina Pénzes, Boglárka Hurják, Éva Katona, Gergely Becs, József Balla, László Muszbek

**Affiliations:** 1 Division of Clinical Laboratory Science, Department of Laboratory Medicine, Faculty of Medicine, University of Debrecen, H-4032 Debrecen, Hungary; kpenzes@med.unideb.hu (K.P.); hurjakbogi@gmail.com (B.H.); ekatona@med.unideb.hu (É.K.); 2 Department of Nephrology, Faculty of Medicine, University of Debrecen, H-4032 Debrecen, Hungary; becsg@belklinika.com (G.B.); balla.jozsef@med.unideb.hu (J.B.)

**Keywords:** α_2_-plasmin inhibitor, end-stage renal disease, factor XIII, fibrinogen, hemodiafiltration, hemodialysis

## Abstract

Hemostasis disorder in patients with end-stage renal disease (ESRD) is frequently associated with bleeding diathesis but it may also manifest in thrombotic complications. Analysis of individual coagulation and fibrinolytic factors may shed light on the background of this paradox situation. Here we explored components essential for fibrin formation/stabilization in ESRD patients being on maintenance hemodiafiltration (HDF) or hemodialysis (HD). Pre-dialysis fibrinogen, factor XIII (FXIII) antigen concentrations and FXIII activity were elevated, while α_2_-plasmin inhibitor (α_2_PI) activity decreased. The inflammatory status, as characterized by C-reactive protein (CRP) was a key determinant of fibrinogen concentration, but not of FXIII and α_2_PI levels. During a 4-h course of HDF or HD, fibrinogen concentration and FXIII levels gradually elevated. When compensated for the change in plasma water, i.e., normalized for plasma albumin concentration, only FXIII elevation remained significant. There was no difference between HDF and HD treatments. Individual HDF treatment did not influence α_2_PI activity, however after normalization it decreased significantly. HD treatment had a different effect, α_2_PI activities became elevated but the elevation disappeared after normalization. Elevated fibrinogen and FXIII levels in ESRD patients might contribute to the increased thrombosis risk, while decreased α_2_PI activity might be associated with elevated fibrinolytic potential.

## 1. Introduction

End-stage renal disease (ESRD) is the final outcome of a variety of chronic kidney diseases, which requires hemodiafiltration (HDF)/hemodialysis (HD) treatment or kidney transplantation for survival. HD removes low molecular weight uremic toxins simply by diffusion, while HDF, although it uses the same type of dialyzer with the same pore size as HD, employs convective transport resulting in the excretion of small to mid-size toxic proteins, as well [1,2]. In general, ESRD is considered to be associated with risk of bleeding partly due to impaired platelet function [3,4,5,6,7]. However, paradoxically, thrombotic manifestations involving both the arterial and the venous side are also rather common in this patient group [8,9,10,11,12]. Such thrombotic complications include ischemic stroke, myocardial infarction, peripheral artery occlusion, deep vein thrombosis, pulmonary embolism and vascular access thrombosis. The coincidence of hemorrhagic diathesis and thrombotic tendency suggests a complex imbalance in the clotting system, platelet function and changes in the fibrinolytic system also contribute to the hemostatic alterations [13,14]. Individual variations in the conditions and treatment of ESRD patients might contribute to the variability of clinical hemostatic symptoms. General functional tests and determination of various hemostatic parameters have been both used to characterize the hemostatic balance of ESRD patients and explore the pathomechanism leading to altered hemostasis. Concerning the clotting system investigations mainly targeted its activation leading to increased thrombin generation. In ESRD patients undergoing regular hemodialysis markers of thrombogenesis including thrombin-antithrombin complex, prothrombin fragment 1.2, D-dimer, fibrinopeptide A, and factor VIII became elevated, suggesting intravascular activation of the coagulation cascade [15,16].

In the present study we were interested how the constituents of the final stage of clotting cascade are influenced by ESRD and its treatment modality. Fibrinogen, factor XIII (FXIII) and α_2_-plasmin inhibitor (α_2_PI) are the key player in the formation of fibrin clot and its stabilization. Thrombin generated as the end-result of activated clotting system transform fibrinogen into fibrin monomers by cleaving off fibrinopeptides A and B from the parent molecule. Fibrin monomers spontaneously polymerize to become fibrin clot insoluble in plasmatic conditions. FXIII is of tetrameric structure consisting of two potentially active A subunits (FXIII-A) and two protective/inhibitory B subunits (FXIII-B). Thrombin removes an activation peptide of 37 amino acids from the N-terminus of FXIII-A and in the presence of Ca^2+^ the truncated FXIII-A assumes an enzymatically active configuration. Active FXIII (FXIIIa) is a transglutaminase that cross-links fibrin α-, and γ-chains by isopeptide bonds and α_2_PI, the main fibrinolysis inhibitor, to fibrin α-chains. This way it makes fibrin resistant to the shear stress of circulating blood and to proteolytic breakdown by the fibrinolytic system (reviewed in [17,18,19,20,21,22]). Sporadic results on changes of fibrinogen, FXIII and α_2_PI in ESRD have been reported [6,23,24,25,26,27,28,29]; however, comprehensive analysis of these constituents in ESRD is still missing.

The aim of the present study was to explore novel aspects concerning components important in fibrin formation and stabilization of the fibrin clot in ESRD patients. We determined fibrinogen and FXIII antigen concentrations, FXIII and α_2_PI activities in ESRD patients and compared the effect of HDF and HD treatments. It was also investigated if inflammation, tested by C-reactive protein (CRP) measurement, influenced these parameters, and if fibrinogen concentration was associated with other investigated parameters. The effects of further variables were also studied, like patient’s age, gender, length of dialysis treatment. While in the first part of the study, the long-term combined effects of ESRD and its treatment by HDF or HD were investigated, in the second part the short-term effect of an actual HDF or HD treatment was studied. In the latter case, changes of fibrinogen, FXIII and α_2_PI levels were monitored during the four-hour HDF and HD treatments. To take the decrease in plasma water during the dialysis treatments and its effect on protein concentrations into account, the above parameters corrected for albumin concentration were also calculated.

## 2. Results

### 2.1. Plasma Fibrinogen and CRP Concentration of ESRD Patients

**Figure 1:**
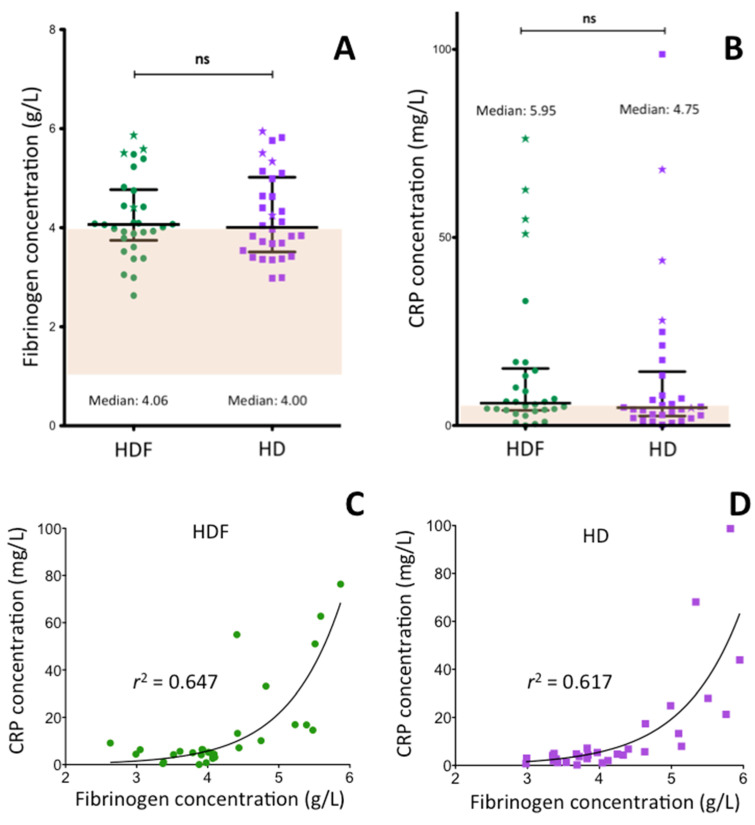
Fibrinogen concentration, C-reactive protein (CRP) level and their relationship in patients with end-stage renal disease. (**A**) Fibrinogen concentration and (**B**) CRP level in patients being on permanent HDF treatment and after switching to HD treatment for two weeks. Longer horizontal lines demonstrate median values (numerical values are also shown), shorter horizontal lines depict the upper and lower limits of interquartile range. The four outliers with CRP level higher than 50 mg/L in the HDF group are labelled by asterisk in both (**A**,**B**). (**C**,**D**) demonstrate the relationship between CRP level and fibrinogen concentration in HDF treated patients and after HD treatment, respectively. CRP: C-reactive protein, HDF: hemodiafiltration, HD hemodialysis, ns: not significant, *r*^2^: coefficients of determination.

In 56.6% of ESRD patients being on HDF treatment fibrinogen concentration was above the reference interval (1.5–4.0 g/L), mean fibrinogen concentration was 4.21 ± 0.82 g/L (Figure 1A). Modality switching from HDF to HD treatment did not influence the fibrinogen concentration significantly. In 50.0% of HD-treated patients it remained above the reference interval, mean: 4.23 ± 0.87 g/L. It is to be noted that even fibrinogen values that felt in the reference interval concentrated predominantly in the upper half of the interval.

As fibrinogen is an acute phase protein and ESRD patients frequently suffer from inflammatory conditions, the concentration of CRP, an excellent marker of inflammatory reactions, was also measured (Figure 1B) and its correlation with fibrinogen concentration (Figure 1C,D) was evaluated. In 56.6% of the patients on HDF treatment, the CRP level was above the upper limit of reference interval (females: 4.6 mg/L, males: 5.2 mg/L). The median CRP plasma concentration of ESRD patients was 5.95 mg/L, with interquartile range (IQR): 4.02–15.15 mg/L and there were a few outliers. The CRP level of four HDF treated patients was above 50 mg/L, due to acute inflammatory conditions. Their results are depicted by asterisks not only in Figure 1B, but also in figures demonstrating the results obtained for other investigated parameters. In spite of the few individual changes during the two-week period of HD treatment, the CRP levels, in general, did not change significantly. In 53.0% of the patients it remained above the upper limit of reference interval (median: 4.75 mg/L, IQR: 2.52–14.33 mg/L). As shown in Figure 1C,D fibrinogen concentration was an exponential function of CRP level. The high coefficients of determination (*r*^2^: 0.647 and 0.671 in case of HDF and HD treated patients, respectively) demonstrate significant correlation (*p* < 0.001) between the inflammatory condition and fibrinogen levels. Neither the length of HDF treatment period (*r*^2^: 0.018, *p* = 0.590) or the age of patients (*r*^2^: 0.035, *p* = 0.267) correlated significantly with the fibrinogen levels (not shown in Figure 1). There was no significant difference between females and males in the pre-dialysis fibrinogen and CRP concentrations.

### 2.2. Factor XIII Activity and Antigen Concentration in the Plasma of ESRD Patients

**Figure 2:**
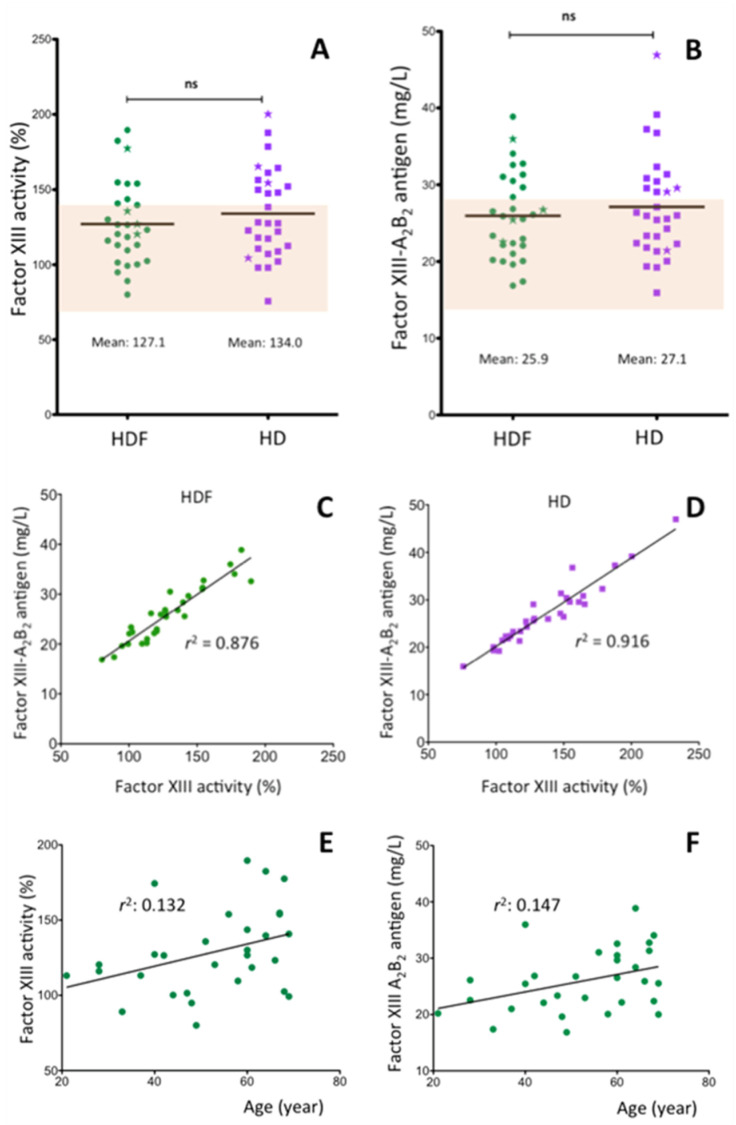
FXIII levels in the plasma of ESRD patients. Plasma FXIII activity (**A**) and antigen concentration (**B**) of patients being on chronic HDF treatment or after two weeks of HD treatment. (**C**) and (**D**) correlation of the results obtained by activity measurement and immuno-assay; (**E**,**F**) correlation of FXIII levels with the age of ESRD patients. HDF: hemodiafiltration, HD hemodialysis, ns: not significant, *r*^2^: coefficients of determination. The asterisks on (**A**,**B**) represent the four patients with the highest CRP levels.

A key component of the last stage of coagulation cascade is FXIII which circulates in association with fibrinogen and in activated form, it works on fibrin by cross-linking fibrin chains and α_2_PI to fibrin. In the following experiments, we investigated whether the concentration of FXIII and α_2_PI which are essential for maintaining normal hemostasis and are involved in the pathomechanism of thrombotic diseases, changes in ESRD. FXIII was measured both by functional test (Figure 2A) and antigen assay (Figure 2B). Similar results were obtained by the two measurement techniques as it was demonstrated by the coefficients of determination both in the HDF (*r*^2^: 0.876, *p* < 0.001) and in the HD (*r*^2^: 0.916, *p* < 0.001) group (Figure 2C,D). In the HDF group the mean FXIII activity was 127.1 ± 27.3% and the mean FXIII antigen concentration was 25.9 ± 5.6 mg/L (Figure 2A,B). Two weeks of hemodialysis treatment did not change significantly the FXIII values (FXIII activity: 134.0 ± 29.8%, FXIII antigen: 27.1 ± 6.7 mg/L). The means in all cases were in the upper tertile of the reference interval. In the HDF group 27% of individual FXIII activity values and 33% of FXIII antigen values exceeded the upper limit of the reference interval. In the HD group 43% (activity) and 40% (antigen) were above the reference interval. These results indicate that in ESRD patients, as compared to healthy individuals, FXIII concentration becomes elevated. Both FXIII activity (*r*^2^: 0.132, *p* = 0.061) and antigen (*r*^2^: 0.147, *p* = 0.070) showed weak nonsignificant correlation with the patients’ age (Figure 2E,F). The length of HDF treatment did not correlate with FXIII levels (*r*^2^: 0.004, *p* = 0.769 for FXIII activity and *r*^2^: 0.003, *p* = 0.272 for FXIII antigen). Neither fibrinogen nor CRP concentration influenced FXIII activity or antigen levels significantly in HDF treated patients (*r*^2^: 0.001 and <0.001 for fibrinogen, *r*^2^: 0.007 and 0.004 for CRP). Results of the latter parameters are not shown in Figure 2. There was no gender-specific difference in the FXIII activity/concentration of HDF- or HD-treated patients.

### 2.3. α_2_-Plasmin Inhibitor Activity in the Plasma of ESRD Patients

**Figure 3:**
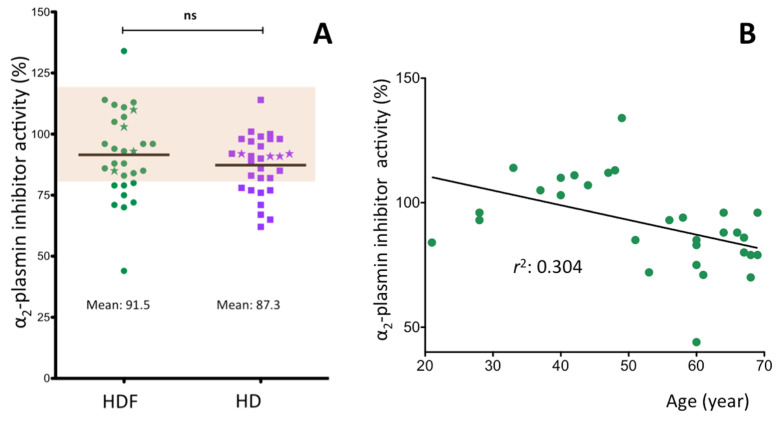
α_2_-plasmin inhibitor activity in ESRD patients. (**A**) patients being on chronic HDF treatment or after switching to HD treatment for two weeks. (**B**) correlation of α_2_-plasmin inhibitor activity with patients’ age. The coefficient of determination (*r*^2^) is also shown in the figure. The asterisks on Figure A represent the four patients with the highest CRP levels.

In contrast to fibrinogen and FXIII, α_2_PI activity of ESRD patients tended to be lower than in healthy individuals (Figure 3A). The mean value both in the HDF and HD groups was in the lowest tertile (91.5 ± 17.7% and 87.3 ± 11.9%). 23% of HDF patients and 27% of HD patients demonstrated α_2_PI activity below the lower limit of the reference intervals. The age of ESRD patients (Figure 3B) negatively correlated with this parameter (*r*^2^: 0.304, *p* = 0.002), while the length of HDF treatment was without effect (*r*^2^: <0.001). Actual inflammation, as detected by lack of correlation with CRP levels did not influence α_2_PI activity. No significant difference of α_2_PI activity was observed between male and female ESRD patients.

### 2.4. Changes of Plasma Fibrinogen Concentration during Hemodiafiltration and Hemodialysis Treatments of ESRD Patients

**Figure 4:**
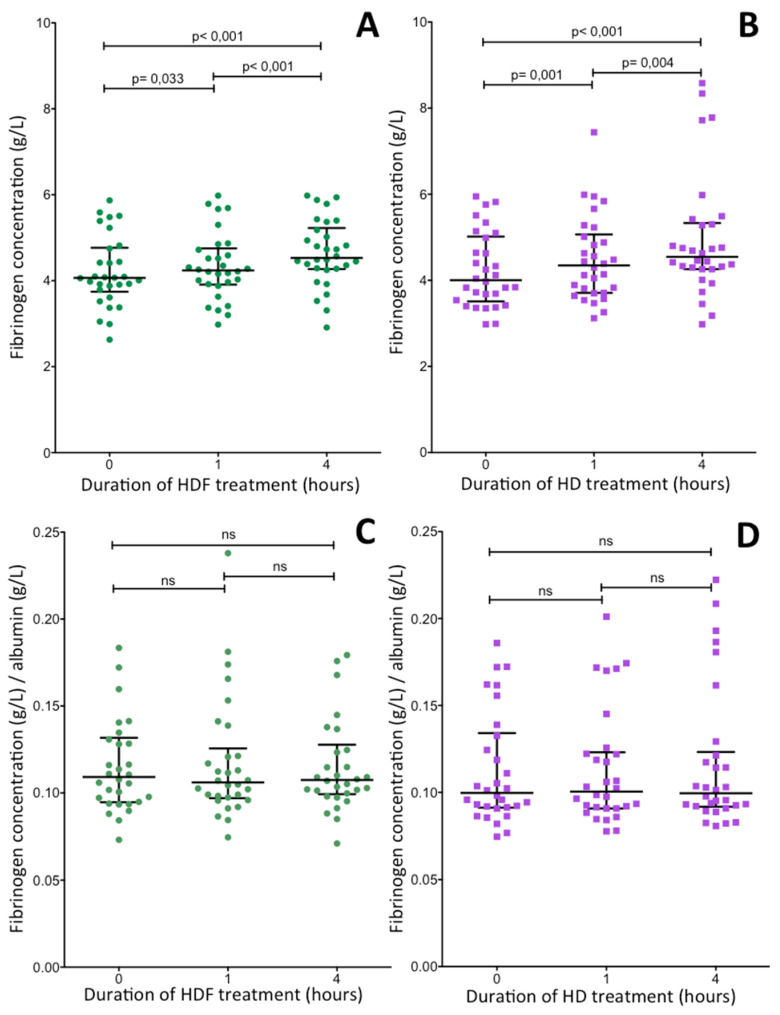
Changes of plasma fibrinogen concentration in ESRD patients during a 4-h HDF (**A**,**C**) or HD (**B**,**D**) treatment. The two upper figures demonstrate fibrinogen concentrations not corrected for albumin (**A**,**B**), while the two lower figures show fibrinogen concentrations corrected for albumin (**C**,**D**). Long and short horizontal lines in the figures represent median values and limits of interquartile ranges, respectively. Significant differences between results obtained before and after treatments are indicated by the p values above thin horizontal lines.

The above results demonstrate the long-term effect of HDF and HD treatments on three key terminal phase components of the hemostasis system. A further interesting question was whether these parameters change during the four-hour individual HDF or HD treatment. As the relation of plasma water and plasma proteins significantly changes during the four-hour treatments, such changes were monitored by the determination of albumin concentrations and results of hemostasis parameters corrected for albumin concentration were also calculated. As expected, albumin concentration gradually elevated during the 4-h HDF and HD treatments. In HDF treated patients, pre-dialysis albumin concentration (median: 37 g/L, IQR: 35–40 g/L) became significantly elevated after 4-h HDF treatment (median: 42 g/L, IQR: 38–44 g/L, *p* < 0.001). In the HD group a similar elevation was observed (pre-dialysis median albumin concentration: 39 g/L, IQR: 36–41 g/L; post-dialysis median albumin concentration: 44 g/L, IQR: 40–48 g/L, *p* < 0.001). Similarly, plasma concentration of fibrinogen also became significantly elevated during the four-hour treatments (Figure 4A,B). When corrected for albumin concentration the differences between the fibrinogen levels measured before and after the 4-h treatment disappeared (Figure 4C,D), i.e., the measured elevations were due to the relative decrease of plasma water and not to the elevated amount of fibrinogen in the plasma.

### 2.5. Changes of Plasma FXIII Activity during Hemodiafiltration and Hemodialysis Treatments of ESRD Patients

**Figure 5:**
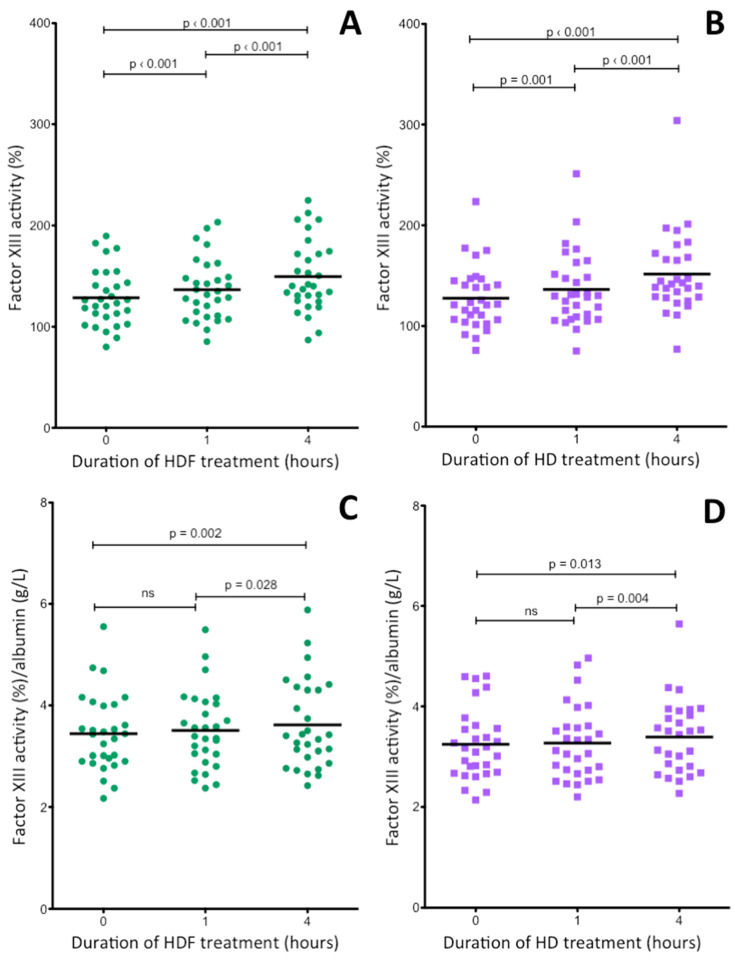
Changes of plasma FXIII activity in ESRD patients during a 4-h HDF (**A**,**C**) or HD (**B**,**D**) treatment. The upper two figures show FXIII activities not corrected for albumin (**A**,**B**), while FXIII activities corrected for albumin concentration are shown in the two lower figures (**C**,**D**). Horizontal lines represent mean FXIII activities. Significant differences between results obtained before and after treatments are indicated by the p values above thin horizontal lines.

In case of FXIII the situation was different (Figure 5). FXIII activity noncorrected for albumin also showed marked elevation during both HDF and HD treatments (Figure 4A,B), however, a statistically significant elevation still remained after correction (Figure 4C,D). The change of FXIII antigen levels was identical to the changes in FXIII activity (not shown).

### 2.6. Changes of Plasma α_2_-Plasmin Inhibitor Activity during Hemodiafiltration and Hemodialysis Treatments of ESRD Patients

**Figure 6:**
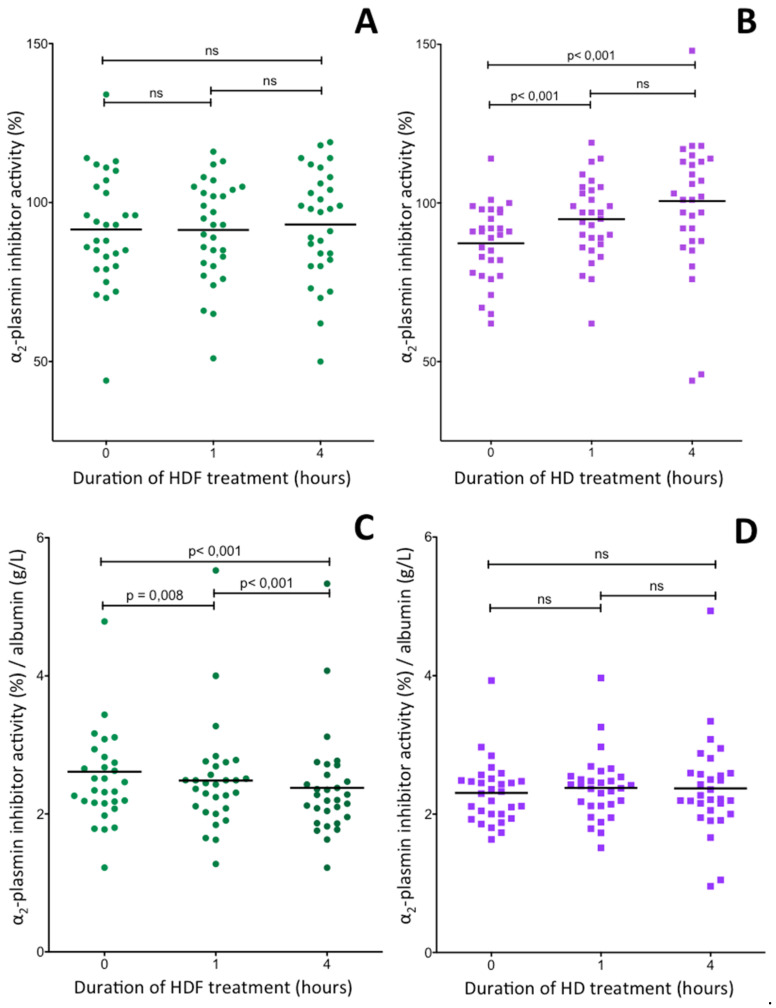
Changes of plasma α_2_PI activity in ESRD patients during a 4-h HDF (**A**,**C**) or HD (**B**,**D**) treatment. The upper two figures show α_2_PI activities not corrected for albumin (**A**,**B**), while α_2_PI activities corrected for albumin concentration are shown in the two lower figures (**C**,**D**). The horizontal lines represent mean activities. Significant differences between results obtained before and after treatments are indicated by the p values above thin horizontal lines.

Interestingly, as opposed to changes in noncorrected fibrinogen and FXIII levels noncorrected α_2_PI activities (Figure 6A) remained the same during the 4-h HDF treatment. This finding suggested that HDF treatment decreased α_2_PI level by some mechanisms and the elevation which could have been caused by the decrease of plasma water was counteracted the decrease in the α_2_PI level. Indeed, when α_2_PI activity was normalized to albumin concentration its decrease became evident (Figure 6C). As opposed to HDF treatment, the effect of decreased plasma water due to HD treatment resulted in the elevation of α_2_PI activities (Figure 6B). Like in the case of fibrinogen concentration (Figure 4D), the elevations disappeared when the results were normalized to plasma albumin concentration (Figure 6D).

## 3. Discussion

The aim of the present study was twofold: First, to explore the effect of long-term hemodialysis treatment on the activity/concentration of fundamental components of the final stage of coagulation cascade in ESRD patients and to compare the two hemodialysis modalities, HDF and HD in this respect. Second, to study their short-term effect, i.e., to monitor individual changes during the four-hour dialysis regimens. The three components selected for the study, fibrinogen, FXIII and α_2_PI, work together in determining the amount and quality of fibrin clot and its fate in the circulation. It is to be noted that the duration of HD treatment was only two weeks as opposed to the several months or years of HDF treatment. This is a limitation of the study concerning the long-term effect; however, such a study plan made it possible to switch modalities and use the same cohort of ESRD patients for testing the effect of both treatments on the same groups of individuals. Another limitation is the relatively low number of patients enrolled for the study. This is partly due to the exclusion of ESRD patients with co-morbidities that were suspected to influence the plasma level of investigated hemostasis parameters independently. Still, it is realized that further investigations on a higher number of patients would strengthen the conclusion.

Fibrinogen is the precursor protein of fibrin clot and beside its fundamental role in hemostasis it also plays an important role in wound healing. Its expression is controlled by the activity of promoters which contain binding sites for hepatocyte transcription factors, including proteins which influence fibrinogen transcription in response to acute-phase inflammatory stimuli [30]. Elevated fibrinogen concentration is an independent risk factor for atherothrombotic diseases like hemorrhagic stroke and ischemic heart disease [31,32,33,34]. Most studies reported elevated pre-dialysis fibrinogen concentration in ESRD patients on chronic HD treatment [24,27,28,35,36,37], although in part of them the elevation did not reach the level of statistical significance [6,38]. Our results (Figure 1A) also demonstrate considerable elevation of fibrinogen concentration in pre-dialysis samples; in 56.6% HDF patients and in 50.0% of HD patients plasma fibrinogen was above the reference interval. These values agree with those of Zoccali et al., who reported 56% pre-dialysis fibrinogen values being above the reference interval [28]. There was no significant difference between the effect of the two modalities. In case of both modalities, pre-dialysis fibrinogen levels and CRP levels were closely associated, and the high coefficients of determinations suggest that at least in part increased synthesis of acute phase proteins is responsible for the elevated fibrinogen levels (Figure 1C,D). Other investigated variables, like the length of dialysis treatment, age and gender did not seem to influence pre-dialysis plasma fibrinogen concentration.

The pre-dialysis values are determined by longer lasting effects on fibrinogen synthesis, the acute effect of a single dialysis treatment is very likely influenced by other fast acting factors. Derosa et al. also investigated the acute effect of single HDF or HD treatment in ESRD patients on fibrinogen concentration [39]. In patients with type 2 diabetes, HDF but not HD decreased post-dialysis fibrinogen concentrations. In euglycemic subjects, the decrease was not statistically significant. We observed a gradual elevation of fibrinogen levels during HDF as well as HD treatment (Figure 4A,B). These elevations were likely associated with the loss of plasma water during dialysis; fibrinogen concentrations corrected for albumin level failed to increase during dialysis treatments (Figure 4C,D).

Sporadic studies addressed FXIII levels in a few ESRD patients being on maintenance hemodialysis treatment. FXIII activity was determined by functional assay only in a single case [25], and in three cases FXIII-A antigen concentration was measured [23,24,25]. Normal pre-dialysis FXIII activity and FXIII-A antigen was reported by Kolb et al. [25], while significantly elevated FXIII-A concentrations were observed in two other studies [23,24]. We determined FXIII activity and the concentration of FXIII-A_2_B_2_ complex in parallel and both parameters were elevated in this patient group. In theory, FXIII activity and FXIII-A antigen could be influenced by proteolytic degradation of FXIII-A and free circulating elastase released by polymorphonuclear leukocytes primed during hemodialysis [40] is capable of degrading FXIII-A [41]. FXIII-A antigen assays could also measure degradation products. For this reason, we selected an immunoassay that measures the complex FXIII tetramer with full activity. The close correlation of FXIII activity and FXIII-A_2_B_2_ antigen (Figure 2C,D) suggest that fully active intact FXIII was measured by the immunoassay. During the 4-h HDF and HD treatments, the FXIII activity became significantly elevated (Figure 5A,B). However, as opposed to fibrinogen levels after correction for albumin concentration FXIII activities still remained elevated although the level of significance somewhat decreased. This finding suggests that the decrease of plasma water itself does not explain the elevated FXIII levels and further studies are needed to reveal the mechanism of FXIII elevation in ESRD patients being on hemodialysis treatment. Kolb et al. demonstrated a decrease in plasma FXIII activity, but not in FXIII-A antigen level during HD treatment [25]. However, heparin introduced in the course of HD treatment into the plasma inhibited thrombin used for FXIII activation in activity measurements and resulted in underestimation of FXIII activity. The Technoclone kit we used for FXIII activity measurement contains polybrene that neutralized the effect of heparin.

The two types of FXIII subunits are originated from different cellular sources. FXIII-B is synthesized by hepatocytes, while FXIII-A is present in platelets, monocytes and macrophages [20]. A recent study identified resident macrophages as the major source of circulating FXIII-A [42]. In the plasma the overwhelming majority of FXIII-A subunit forms noncovalent tetrameric complex with FXIII-B, which is present in excess [43]. The formed A_2_B_2_ complex then circulates in association with fibrinogen [44,45,46]. In healthy individuals, age and fibrinogen concentration significantly correlated with FXIII activity and FXIII-A_2_B_2_ antigen concentration [47]. In ESRD patients there was also a weak correlation between FXIII values and the patients’ age (Figure 2E,F), but very likely due to the relatively low patients’ number the p values were only close to the level of significance. It is interesting that in the healthy population there was relatively weak, but statistically significant correlation between FXIII and fibrinogen concentration, while in ESRD patients this association completely disappeared. As opposed to fibrinogen, FXIII, is not an acute phase protein, which is also demonstrated by the lack of association between FXIII and CRP levels. It is suspected that due to the robust influence of inflammatory status on the fibrinogen concentration the association between fibrinogen and FXIII concentration became concealed in ESRD patients.

It is an intriguing question if elevated FXIII level in ESRD patients represents a risk of atherothrombotic or thromboembolic diseases. This presumption has been tested in a few studies, but no general conclusion could be drawn, so far. There are indications that it might confer increased thrombotic risk to certain subpopulations. For instance, elevated FXIII levels were associated with an increased risk of myocardial infarction and peripheral artery disease in females, but not in males [48,49]. Young patients with the history of myocardial infarction had elevated FXIII activity and antigen levels [50]. Similarly, to atherothrombotic diseases elevated FXIII activity and complex antigen levels conferred an increased risk of venous thromboembolism to females, which remained significant after adjustment for a number of variables [51]. In contrast, only a weak association was observed in the male population. A new clinical study involving larger number of patients could shed light on the association of elevated FXIII levels with thromboembolic and atherothrombotic complications.

α_2_PI (also called α_2_-antiplasmin) is a 70 kDa glycoprotein that exists in the plasma in multiple forms due to genetic variation and posttranslational proteolytic modifications (reviewed in [52]). It inhibits circulating plasmin and if cross-linked to fibrin by FXIIIa, it protects fibrin from fibrinolytic degradation [20,52]. Its importance in hemostasis is clearly shown by the severe bleeding diathesis of patients with congenital α_2_PI deficiency [53]. There are only a few studies on the association of α_2_PI with thrombotic diseases. A clinical study demonstrated that elevated α_2_PI levels were independently associated with the risk of myocardial infarction in men [54]. Age-, sex- and race-adjusted hazard ratios for the highest and lowest quartiles demonstrated a protective effect of elevated α_2_PI against ischemic stroke in the ARIC (Atherosclerosis Risk in Communities) study [55]. However, after multivariate analysis including other clinical variables the standardized hazard ratio did not remain significant. In a mouse model elevation of α_2_PI levels in the blood worsened the consequences and the outcome of microvascular brain thrombosis and impaired the benefit of thrombolytic treatment, while decrease or lack of α_2_PI had the opposite effect [56]. Furthermore, elevated α_2_PI impaired the effectivity of thrombolysis induced by tissue plasminogen activator. Similar findings were reported for mice with acute pulmonary embolism [57].

We found only three studies in which α_2_PI activity was measured in ESRD patients being on HD [24,26,58]. Only one of them reported post-dialysis values and the effect of HDF has not been investigated. Our results, i.e., the mean pre-dialysis α_2_PI activities were in the lowest tertile of the refence interval and a significant number of the results was below the reference interval. In this respect there was no significant difference between HDF and HD treatments. These results together with those of two earlier studies [24,58] suggest that long term HDF or HD treatment of ESRD patients decreases the plasma concentration of active α_2_PI. Monitoring the short-term effect of HDF and HD treatments revealed an interesting difference between the two modalities. α_2_PI activities not corrected for plasma albumin concentration did not change during the course of an HDF treatment, while they became significantly elevated after a 4-h HD treatment. Correction for albumin considerably altered the situation. In HDF treated patients α_2_PI activities normalized to albumin concentration decreased, while the elevation observed in HD treated patients disappeared. This finding suggests that the mechanisms operating in HDF treated patients and resulting in decreased α_2_PI activity normalized to albumin do not function in case of HD treatment. It is to be noted that Vasiri et al. also found elevated α_2_PI activities after HD treatment; however, a change in plasma water during dialysis was not taken into account in their study [24].

## 4. Materials and Methods

### 4.1. Patients and Blood Sampling

Thirty ESRD patients (15 females and 15 males) on chronic dialysis treatment were enrolled for the study. Their age was in the range of 18–70 years (median: 57 years, interquartile range: 41.5–64.5 years). Patients’ data are summarized in the Supplementary Table. Patients with diabetes mellitus, malignant disease and hematological or hemostasis disorders were excluded. At the time of recruiting, the ESRD patients were on the HDF treatment—the preferred modality in our dialysis center—for at least twelve months (median period of HDF treatment: 54 months, interquartile range: 33–108 months). During the investigations there was a modality change, for two weeks the patients were switched to HD treatment, after which the HDF treatment was reinstalled. The dialysis sessions were provided three times a week and one session lasted for effective 240 min for both treatment modalities. Further details of HDF and HD treatments have been described in a previous publication [5] and also in the Supplementary Materials. Before, one and four hours after the actual HDF or HD treatment peripheral blood samples were drawn from the efferent line port into vacutainer tubes (Becton Dickinson, Franklin Lakes, NJ, USA) containing 0.109 mol/L sodium citrate. Plasma from citrated blood was separated by centrifugation (1500× *g*, 20 min, 4 °C), aliquots of plasma samples were stored at −70 °C until further analysis. The study protocol fully complied with the Declaration of Helsinki and it was approved by the Ethics Committee of the University of Debrecen (approval code: HRB/052/00511-2; approved on 12.11.2013). Informed consent was obtained from the participating patients.

### 4.2. Laboratory Measurements

FXIII activity was determined by the ammonia release assay with blank correction [59] using TECHNOCHROM^®^ reagent kit (Technoclone, Vienna, Austria) on Modular EVO P800 analyzer (Roche/Hitachi, Mannheim, Germany). The complex FXIII antigen (FXIII-A_2_B_2_) concentration was measured by ELISA [43]. Berichrom chromogenic antiplasmin test (Siemens, Marburg, Germany) was used for the determination of α_2_PI activity. Plasma fibrinogen concentration was measured by the Clauss method using the Labexpert LX fibrinogen assay kit (Labexpert Ltd., Debrecen, Hungary). High sensitivity CRP (HS) reagent from Diagnosticum Ltd. (Budapest, Hungary) was used for the measuring CRP concentration. Serum albumin concentration was measured by the bromocresol purple absorption method on Roche/Hitachi cobas c system.

### 4.3. Statistical Analysis

Statistical analysis of the results was performed by the Statistical Package for Social Science (SPSS, 22.0, Chicago, IL, USA) and by GraphPad Prism version 5.0 (GraphPad Software, Inc., San Diego, CA, USA). Distribution of the data was evaluated by the Kolmogorov Smirnov test. GraphPad Prism software was used for the construction of the figures. In case of nonparametric distribution, the results measured after HDF treatment and following the two-week HD treatment were compared by related-samples Wilcoxon signed rank test, while in case of parametric distributions related sample *t*-test was used. Differences in tested parameters measured before, one and four hours after the initiation of individual HDF and HD treatment were compared by Friedman test (nonparametric distribution) or by repeated measure ANOVA test (parametric distribution). The association between various parameters were analyzed by Spearman rank correlation.

## 5. Conclusions

In ESRD patients being on chronic HDF or HD treatment fibrinogen, FXIII antigen concentrations and FXIII activity were elevated, while α_2_PI activity decreased. As demonstrated by the significant correlation between the CRP level and fibrinogen concentration, the inflammatory status was a key determinant of fibrinogen concentration, but not of FXIII and α_2_PI levels. During a 4-h course of individual HDF or HD treatment, fibrinogen concentration and FXIII levels gradually elevated. However, when normalized for plasma albumin concentration, only the FXIII elevation remained significant. As opposed to fibrinogen and FXIII, the effect of individual HDF and HD treatments on plasma α_2_PI activity was different. After normalization to albumin significantly decreased α_2_PI activity was measured during HDF treatment. In HD treated patients, α_2_PI activities became elevated but the elevation disappeared after normalization. Elevated fibrinogen and FXIII levels in ESRD patients might contribute to the increased thrombosis risk, while decreased α_2_PI activity might be associated with elevated fibrinolytic potential.

## Supplementary Materials

The following are available online at https://www.mdpi.com/1422-0067/21/22/8426/s1.

## Abbreviations

α_2_PI	α_2_-plasmin inhibitor
CRP	C-reactive protein
ESRD	end-stage renal disease
FXIII	blood coagulation factor XIII
FXIII-A	FXIII A subunit
FXIII-B	FXIII B subunit
HD	hemodialysis
HDF	hemodiafiltration
IQR	interquartile range
