# Supplementary material for: Terminal Phase Components of the Clotting Cascade in Patients with End-Stage Renal Disease Undergoing Hemodiafiltration or Hemodialysis Treatment
Source: Int J Mol Sci. 2020 Nov 10;21(22):8426. doi: 10.3390/ijms21228426 (PMC7697748; doi:10.3390/ijms21228426)
Supplement: Supplementary file 1 [file ijms-21-08426-s001.pdf]

Supplementary Materials

Patients' data

Supplementary Table

Data of patients with end-stage renal disease being on hemodiafiltration (HDF) and hemodialysis (HD) treatment

Gender	15 females, 15 males	
Age	18-70 years (median: 57 years, IQR: 41.5-64.5 years)	
Co-morbidities that occurred in more than a single patient	Hypertension (24), previous kidney transplantation (9), chronic heart disease (6), autosomal dominant polycystic kidney disease (5), gastro-esophageal reflux disease (3), epilepsy (3), benign prostate hyperplasia (2), Goodpasture-syndrome (2)	
Most frequent medications	Phosphate binder (30), vitamin D with or without Ca sensing receptor agonist (21), vitamin B with or without folic acid (18), antacids (15), statins (14), calcium channel blocker (14), allopurinol (14), beta receptor blocker (14), ACE inhibitor or ATII receptor blocker (12), alpha receptor blocker (12), diuretics (9), alpha and beta receptor blocker combined (6).	
Treatment	HDF	HD
Albumin	median: 37 g/L, IQR: 35-40 g/L	median: 39 g/L, IQR: 36-41 g/L
Total cholesterol	4.34±0.91 mmol/L	4.17±0.82 mmol/L
Parathyroid hormone	75.9±53.6 pmol/L	67.3±42.7 pmol/L
Calcium	2.00±0.26 mmol/L	2.04±0.29 mmol/L
Inorganic phosphate	1.59±0.56 mmol/L	1.58±0.39 mmol/L
C-reactive protein	median: 5.9 mg/L, IQR: 4.0-15.1 mg/L	median: 4.8 mg/L, IQR: 2.5-14.3 mg/L

The number of patients with the specified co-morbidity is shown in parenthesis. Similarly, the number of patients receiving certain medications is also indicated in parenthesis. Additional 19 other drugs, each received by fewer than 5 patients, are not included in the table.

Plasma total cholesterol, parathyroid hormone, calcium and inorganic phosphate values showed normal distributions and they are represented by means ± standard deviations in the Table. The distribution of albumin and C-reactive protein was nonparametric, in these cases medians and IQRs are shown. The high parathyroid hormone values are due to secondary hyperparathyroidism.

Reference intervals of laboratory parameters: albumin: 35-52 g/L; total cholesterol: 2.0-5.2 mmol/L; parathyroid hormone: 1.6-6.9 pmol/L; calcium: 2.10-2.60 mmol/L; inorganic phosphate: 0.80-1.45 mmol/L; C-reactive protein: <4.6 mg/L (females), <5.2 mg/L (males).

Abbreviations: ACE, angiotensin converting enzyme; ATII, angiotensin II; IQR, interquartile range.

Details of dialysis treatments

The dialysis treatments were accomplished by Fresenius 5008S devices (Fresenius Medical Care, Bad Homburg, Germany), and Cordiax FX dialyzers, FX60, FX600 and FX800 respectively, which were matched to patient's weight and blood flow rate as the effectiveness required it. The type of dialyzers remained the same during the study between the modalities. To deliver a successful treatment we had to achieve a Kt/V higher, than 1.3 in hemodialysis and more than 1.4 during hemodiafiltration. Kt/V for each treatment was calculated by the Online Clearance Monitoring Kt/V (OCM Kt/V; Fresenius Medical Care, Bad Homburg, Germany) measurements. The same speed of blood flow (mean 366.1 ± 63.7 mL per minute) and dialysate flow (439.3 ± 76.5 mL per minute) was used in both modalities. The net ultrafiltration during hemodialysis and hemodiafiltration was 2703.6 ± 1118.0 mL per session and 2432.1 ± 1014.4 mL per session, respectively. We started the sampling with hemodiafiltration as it was the preferred modality in our dialysis center. As anticoagulant we used unfractionated heparin during both types of treatments with the same amount of heparin, which was adjusted to the patient's need to avoid blood line coagulation and bleeding from AV-fistulas. During hemodiafiltration we used the Fresenius AutoSub function to achieve a better efficiency, thanks to which the delivered volume of substitution fluid was 23.4 ± 3.8 L per session. The bicarbonate dialysis solution contained the same amounts of solutes for both modalities. The dialysis fluid was manufactured on-line from ultrapure water and consisted of 138 mmol/L sodium, 2 or 3 mmol/L potassium depending on the patient's potassium value, 1.25 mmol/L calcium, 0.5 mmol/L magnesium, and 1 g/L glucose. The substitution fluid was prepared on-line from dialysis solution through another membrane to purify it before infused directly into the blood line. Bicarbonate concentration of dialysis and substitution fluid was adjusted between 28–38 mmol/L to obtain plasma bicarbonate level of 20–22 mmol/L prior to dialysis. The purity and sterility of dialysate/replacement fluids met the criteria of the 8th edition of European Pharmacopoeia (endotoxin level <0.01 EU/mL, bacterial counts <0.1 CFU/mL and any heavy metal ions <0.01 mg/L).
